# Influence of Ca doping in structural, electronic, optical and mechanical properties of Ba_1−x_Ca_x_TiO_3_ perovskite from first-principles investigation

**DOI:** 10.1038/s41598-023-36719-8

**Published:** 2023-06-28

**Authors:** Zahid Hasan, M. Atikur Rahman, Dipta Kumar Das, Hasan Khaled Rouf

**Affiliations:** 1 Department of Electrical and Electronic Engineering, Southeast University, Dhaka, 1208 Bangladesh; 2 Department of Electrical and Electronic Engineering, University of Chittagong, Chittagong, 4331 Bangladesh

**Keywords:** Materials science, Mathematics and computing

## Abstract

Nowadays, perovskite materials are well known for electronics and optoelectronics applications. We have investigated a potential candidate for those applications to compare the applicability in optoelectronics, photorefractive and photovoltaic (PV) devices. The systematic comparative study of the structural, electronic, optical, mechanical, and thermodynamic properties of pure BaTiO_3_ and Ca doped BaTiO_3_ (Ba_1−x_Ca_x_TiO_3_ where x = 0.125, 0.25, 0.375, 0.500, 0.625) perovskite have been carried out using first-principles and density-functional-theory calculations as recently this material was mostly experimented. The measured structural parameters from the geometrically optimized structure of cubic BT ceramic compared with the other theoretical values. A crystal phase transition occurs when doping content x = 0.25. The electronic band structure shows that the nature of the bandgap is changed from indirect bandgap to direct bandgap energy at G-point after doping the Ca atom into BaTiO_3_ (BT) crystal. Doping of Ca into BT has led to bandstructure modification including conduction band (CB) shifting toward the higher energy level. Electronic properties have been reported to examine the contribution of different orbitals to the CB and to the valance band (VB). This study investigated the modification of optical properties such as absorption, reflectivity, refractive index, extinction coefficient, conductivity, dielectric function and loss function at the energy range from 0 to 30 eV. The prominent absorption peak and optical energy were observed at the UV light energy region. Based on the optical behavior of the material this theoretical research suggests that the doped BT solution is a suitable candidate for photorefractive and optoelectronic devices. Different elastic constants reveal mechanical stability and the existence of the covalent bond of those compounds. Debye temperature increases with doping content. Hence modification of BaTiO_3_ crystal by Ca atom significantly develop various properties that led it to multifunctional applications.

## Introduction

ABX_3_ formula was introduced as perovskite materials after discovering the first perovskite material CaTiO_3_^1^. In this formula, A and B are positive ions and X is denoted for a negative charge atom means anion. The structure of the unit cell of perovskite is ideally cubic. Here the A site ion is at the corners of the cube cell and B at the body center position and the X is face-centered^2^. The term “perovskite” was named after Russian mineralogist Lev Perovski. From the beginning stage of the discovery of perovskite materials, it has been widely used in sensors, waveguides, memory cells and optical devices^3,4^. Consequently, Perovskite of the form ABO_3_ is considered the most interesting material for optoelectronic devices. In this case, the X term replaces the oxygen ions and is known as perovskite oxide. Oxide based perovskite has the capability to meet the modern revolution in electronics, sensors and industrial areas. Modification of the perovskite material received so much interest from researchers and sensor developers due to their strong chemical and physical modification. The insulating, semiconducting and conducting features of doped perovskite material growing to reach a wide range of applications.

The valuable properties like ferroelectric, piezo-electric, electro-optic and magnetism effects are responsible for the wide range of interest in the modern revolution^5,6^. Most used oxide based perovskite material, BaTiO_3_ (BT) is a novel perovskite material used in versatile applications for its exciting chemical and physical properties. After its discovery in 1941, a practical interest arises among researchers to investigate its various properties such as dielectric, ferroelectric, piezoelectric and optical properties, etc.^7–9^. Basically, it was invented as an alternative to mica for uses like an insulator because of its 8 times greater dielectric value than usual mica. Moreover, the tag of a wide range of applications received for its dominant properties like ferroelectric and piezoelectric which leads the material to use in transducers, Multilayer ceramic capacitors, optical sensors and many others^10,11^. Though BT was invented as a dielectric material that can be used in capacitors and other applications it also has other useful properties like electrical, optical and mechanical properties as well^12^. In the last two decades many dopants have been used to investigate the various properties and to find the optimum composition to use in their respective field. A site dopants like Bi, Li, Sr, Zr, Ca, Ce and many more are used nowadays^13,14^. Further research found all other extraordinary features of that perovskite for why it turned out a good choice in the industry as well as among researchers as a research topic. One of the extraordinary properties of BT such as it can exist in various phases at various temperatures. Depending on the temperature, crystal phases change internally among various forms. Approximately it exists in five crystalline forms that are divided between ferroelectric and paraelectric phases. BaTiO_3_ shows a tetragonal (p4/mm space group) phase at room temperature and cubic (Pm$\overline{3}$m) at higher than the curie temperature because of Ti deviation from oxygen octahedron center. Barium Titanate shows great stability of tetragonal structure at the range of temperature 5 °C to 120 °C and below this range it gradually transit from tetragonal to orthogonal (Amm2) phase. If its temperature reach to the currie temperature (120 °C) cubic phase occurs^15,16^. The phase transition is also depending on the doping level along with the temperature and pressure changes^17,18^.

The tetragonal form is a ferroelectric phase that shows spontaneous polarization. The polarization phenomenon happened without an external electric field in the tetragonal form and enables to control of the electric field for why this mechanism makes BT a suitable element for optoelectronic applications^19^. It is more stable in the cubic form at 120 °C temperature^20^. Basically, cubic and hexagonal refer to the paraelectric phase. Below 120 °C temperature, it exists in three ferroelectric phases, mainly in tetragonal form. So, here cubic form (pm$\overline{3}$m space group) above curie temperature acts like a paraelectric phase and is usually found at high temperatures^21^. Researchers are trying to find new properties and new applications of novel BT ceramics. Their great interest in theoretical investigation helps them to find the approximate behavior of the materials whereas experimental investigation helps to characterize material more accurately. These investigations integrated with the study of ferroelectric, magnetic, optical, electrical and thermal properties. Since there are several inner degrees of freedom of the atoms formatted in the perovskite structure, each of these phases must be optimized^8^. Properties are dependent on their various phase form alongside some factors like grain size, sintering temperature, sample preparation method etc. For example, pure BT has a variation of the dielectric constant at different temperatures and frequencies. But here in our study, we have focused on the change of properties based on doping. A good number of investigations have been done on BT. And now modification of BT has brought great attention to the researchers. BaTiO_3_ is doped with donor atoms and investigated with various properties^22^. Doping brings out a dramatic change in physical behavior as well as modification of properties related to the internal structure of doped BT. Numerous research activities have been done for both pure BT and doped BT till the present due to more feature exploration. Researchers found a fruitful outcome after the incorporation of atoms into BT. As ABO_3_ format of BT perovskite Ba acts as A site, Ti acts as B site and perovskite doping mainly lead by A & B site doping. Some doping improves its electrical properties and ferroelectric properties, some dopants significantly increase its optical properties. The appropriate dopants can leads the material to the highest value in a specific field of application. Many dopants have been used to investigate the improved properties of BT^23–26^. In particular, the elements of group I-III and rare-earth elements from the periodic table have become the most valuable dopants due to their strong change in structural properties i.e. ion size, bond length. Many types of research have been conducted by researchers experimentally along with the theoretical impact on mechanical and electrical properties. Shahid et al*.*^27^ found improved dielectric and ferroelectric properties by incorporating Sr atom into BaTiO_3_ crystal in their experimental work. That’s why for inquiry and modification of the properties provided by pure BT, a doping study is required and also to elaborate its applications in physical life. Tahiri et al.^28^ used Ca and Sr dopants to modify BT where they found a great influence of dopants in optical and electronic properties. In his study, he used ABINIT ab initio software package based on density functional theory (DFT) to obtain the characterization. Computing was performed by generalized gradient approximation (GGA) with the Fritz–Haber-Institute and Perdew–Burke–Ernzerh. Basically, he was comparing the results of doping with the parent samples. Some of the findings are interesting and got my attention. The bandgap energy is much higher which very close to the experimental result of the literature. His study is very close to the present study but in this study, readers can find more interesting information and characteristics. Keswani et al.^29^ investigated Ca and Zr substituted BT with the help of experiments as well as theoretical calculation. They have noticed a good influence of dopants on piezoelectric and ferroelectric properties. Rizwan et al*.*^30^ investigate the optical behavior influenced by La ions on BT. Maldonado^31^ performed DFT calculation on BT ceramic substituted by Ag and La atoms and found appreciative structural properties. There are many experimental investigations with the most available material, Calcium (Ca) powder doped BT ceramic in perovskite, multiferroic material formation. Zahid et al.^32^ reported that the availability of Ca powder on the earth’s surface and its improvement capability of dielectric and multiferroic properties in BT ceramic led this material a great choice nowadays. The improved properties of dielectric and ferroelectrichelps the dopant to use for multifunctional device application such as spintronic device. Sadhana et al.^33^ also reported their investigation on multiferroic properties using Ca doped BT ceramic and found that doped materials have excellent properties than pure BT ceramic. Their reported materials for perovskite phase in multiferroic composite dominate in use of modern semiconductor devices. Hence, we focus to investigate the properties of BT ceramic with Ca atom doping as can compare with experimental literature and to find the suitable content of donor atom where it will be a good candidate for use in optoelectronic and PV materials. Comparing most of the investigations both theoretical and experimental, we have found that donor atoms like Ca can improve the optical properties of BT ceramic perovskite. So, we have investigated theoretically various properties of Ca doping BT ceramic at different contents.

In this paper, we have presented a theoretical investigation on Ca atom doped BT using CASTEP code. Here, First-Principles Density Functional Theory (DFT) study has been performed on A site doping of pure BT ceramic by Calcium (Ca) using the doping formula Ba_1-x_Ca_x_TiO_3_ to examine its effect of changes through investigating structural, electronic and optical properties. Due to the recent trend of research with perovskite and the following literature, we have focused on these three basic properties. We aimed to investigate what changes occur when Ca is doped in different quantities. The purpose of our work is to investigate various properties and compare the data with existing experimental literature, hence finding suitable Ca concentrations where optical properties have been improved. The sections are designed in such a way that a brief background comes with the literature study in the introduction section. Then we will provide an overview of the computational details of the used techniques and parameters. Furthermore, the Result and Discussion section will provide details about investigated properties. Finally, the article will end with conclusions.

## Computational details

**Figure 1:**
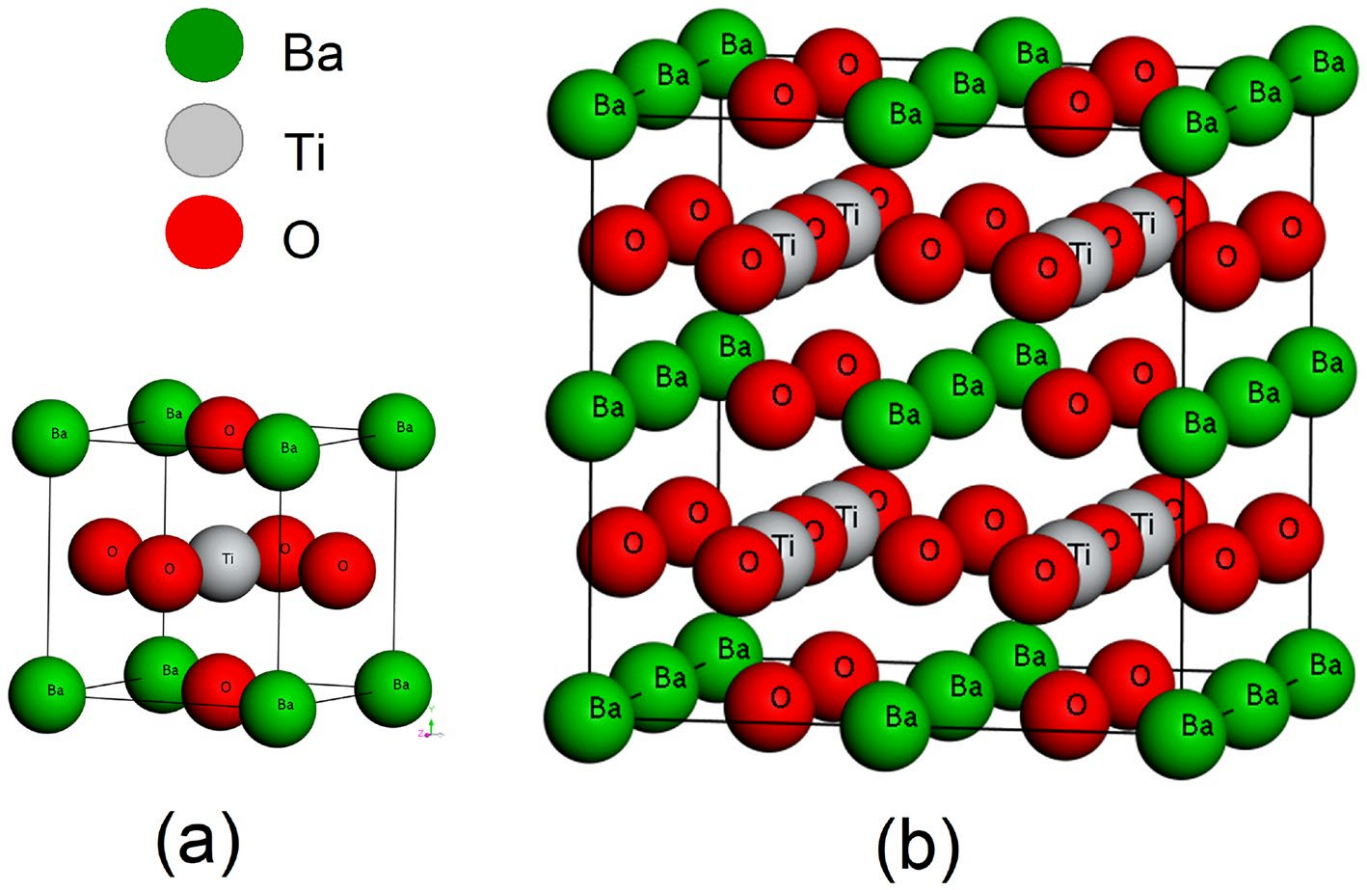
BaTiO_3_ cubic (pm$\overline{3}$m) crystal structure (**a**) Unit cell, (**b**) 2 × 2 × 2 supercell.

**Figure 2:**
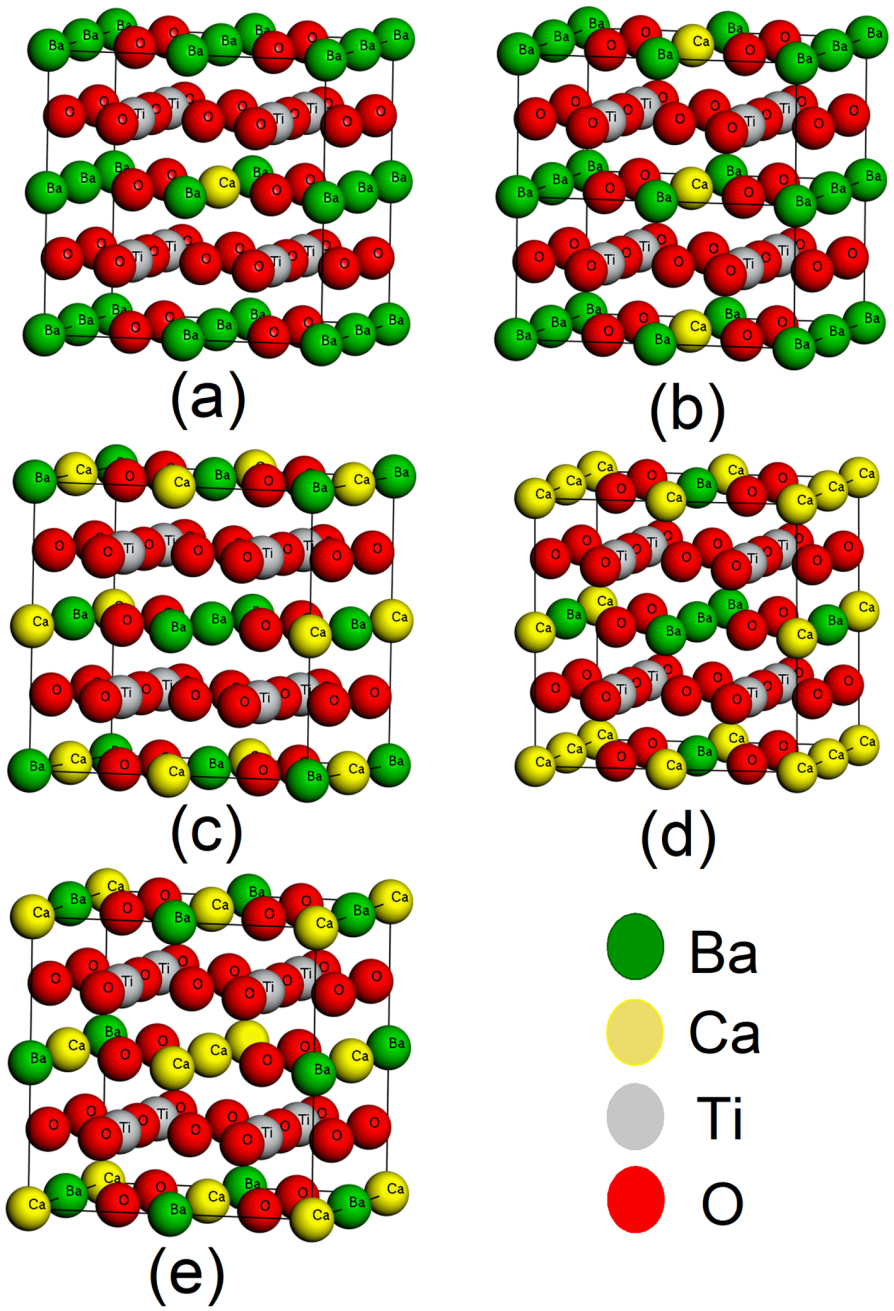
Ba_1−x_Ca_x_TiO_3_ atomics ordering in 2 × 2 × 2 supercells for (**a**) x = 0.125, (**b**) x = 0.25, (**c**) x = 0.375, (**d**) x = 0.50 and (**e**) x = 0.625.

Our presented theoretical investigation has simulated following the pseudopotential DFT-based first-principle method^34,35^ to obtain structural, electrical and optical properties of pure and doped BT oxide perovskite ceramic. Computation based Investigation of properties has been done with Cambridge Serial Total Energy Package (CASTEP)^36,37^ quantum mechanical code which was introduced by Payne along with his colleague in the 1990s to observe various materials properties, especially bandgap and bond lengths investigation. Here, The Generalized Gradient Approximation (GGA) using the Wu-Cohen (WC) functional (GGA-WC)^25,38^ has been used in the CASTEP code package for the calculation of geometrical optimization, all other properties and for exchange–correlation interaction. The symmetrical formation of BT is cubic at the temperature of 120 °C including the pm$\overline{3}$m space group. The structure of a cubic BT unit cell and 2 $\times$ 2 $\times$ 2 supercell has illustrated in Fig. 1a,b respectively. The supercell model of BT 2 $\times$ 2 $\times$ 2 is 8 times greater than the unit cell. The supercell model has been selected for doping the Ca atom. We perform the doping directly using the supercell, where Ba atoms are replaced with Ca dopant. The Illustrated supercell in Fig. 1b) holds 8 Ti atoms inside of 4 unit cells with 24 oxygen atoms and 8 barium atoms out of a total of 40 atoms. Calcium (Ca) atom is doped with respect to Barium (Ba) which means A site doping has been performed in BT perovskite. Doping of Ca has been performed following the formula Ba_1−x_Ca_x_TiO_3_ where (x = 0.125, 0.25, 0.375, 0.500, 0.625). This study is to find the effect of Ca dopant on BT ceramic rather than comparing with the parent sample. So doping concentration is a in lower grade and limited in number. All the doped structure of BT shows in Fig. 2. There are 8 Ba atoms in a unit cell which are being replaced with Ca atoms in such a way that maintain the concentration ‘x’. One Ba atom is replaced with Ca for obtaining a doping concentration x equal to 0.125 (= 1/8) by the direct supercell method illustrated in Fig. 2a. Consequently Fig. 2b–e indicates the doping level of x = 0.25 (2/8), x = 0.375 (3/8), x = 0.50 (4/8) and x = 0.675 (5/8) respectively. The doping procedure is performed by replacing the integer number of Ba atoms with Ca atoms rather than the mixing procedure available in the software package, hence the level of doping concentration. The replacement procedure is done with a supercell of BT ceramic because of its effectiveness. CASTEP calculation and analysis were performed following the density functional theory including the plane-wave pseudopotential approach which guarantees rapid results with good accuracy. The ultrasoft pseudopotential (USP) put forward by GGA-WC has been utilized to the confrontation of electron–ion interaction^30^. The cut of energy was set to 700 eV including k mesh 4 $\times$ 4 $\times$ 4 to perform the irreducible Brillouin zone integrations for geometrical optimization and other simulation. The maximum displacement of 1 × 10^–3^ Å, the maximum stress of 0.05 GPa and the tolerance force per atom of 5.0 × 10^−6^ eV/Å were set.

## Results and discussion

### Structural properties

The general Cubic structural figure of BaTiO_3_ is presented in Fig. 1 as we mentioned in the introduction part. Doping occurs in changes in structural parameters. The measured structural parameters from the geometrically optimized structure of cubic BT 2 × 2 × 2 supercell and Ca atom added 2 × 2 × 2 supercells are presented in Table 1. A little variation of the bond length and bond angle of atoms in the supercell is observed. In a unit cell, Barium (Ba) exists on each corner with Oxygen in the face center position illustrated in Fig. 1a). Ba positioned with fractional coordinates (0, 0, 0), Oxygen with fractional coordinates (0, 0.5, 0.5), (0.5, 0.5, 0), (0.5, 0, 0.5) and Ti with fractional coordinates (0.5, 0.5, 0.5). The position of Titanium (Ti) at the body center into the atom. The coordinate changing of Ti introduced the off-center displacement, δz which indicates the presence of ferroelectric behavior of doped BT perovskite material. The Ti displacement, δz (off-center) presented in Table 1 indicates the presence of spontaneous polarization behavior in the solid solutions. The displacement, δz minus indicates that Ti is down towards the unit cell plane whereas positive indicates the up from the lattice center.

**Table 1:** Variation of the bond length and angle of pure BaTiO_**3**_ and Ca doped BaTiO_3_.

Parameter	Sample: Ba_1−x_Ca_x_TiO_3_					
	x = 0	0.125	0.25	0.375	0.50	0.625
Displacement of Ti (Å)						
δz (off center)	–	0.01520	0.03068	− 0.01566*	− 0.03156	0.01656
Bond length (Å)						
Ba(2)–Ti(1)	3.446	3.456	3.451	3.412	3.405	3.883
Ba(2)–O(3)	2.813	2.818	2.862	2.796	2.766	2.754
Ti(1)–O(3)	1.989	1.996	1.972	1.949	1.924	1.964
Ti(1)–O(5)	–	1.965	2.003	1.980	1.986	1.932
Ca–Ti(1)	–	3.404	3.380	3.394	3.332	3.402
Ca–O(3)	–	2.750	2.750	2.778	2.766	2.781
Bond angle (°)						
Ba(2)–Ti(1)–Ba/Ca(4)	70.528	70.115	70.111	70.961	70.517	70.071
Ba(2)–Ti(1)–Ba/Ca(6)	109.471	109.055	109.685	109.901	110.338	109.009
Ba(2)–Ti(1)–O(3)	54.734	54.617	54.392	54.875	53.859	54.508

*δz minus indicates that Ti is down from center to the unit cell plane whereas positive indicates to the up from the lattice center.

When Ti goes up from its center coordinate material is called positively polarized, polarization changes its direction when Ti goes down from the center coordinate which is known as negatively polarized material. The average bond length and bond angle were measured after geometrical optimization of pure and doped BT and all the data are also gathered in Table 1. The change in bond length and bond angle is due to the lower ionic radius of Ca compared to Ba atoms. Hence, the covalent bond is influenced by Ca doping. In this pure and doped perovskite structure, the octahedral site is occupied by the Ti atom where Ti–O bond length is related to that site and the Ba–O bond lengths are composed of dodecahedral site. The maximum value of relaxed bond lengths of Ba–O and Ti–O are 2.862 Å at x = 0.25 and 1.996 Å at x = 0.125, respectively. These data for pure BT ceramic are in excellent accord with other theoretical values^39,40^. In addition, lattice parameters, volume and symmetry space of BT and Ca doped BT (BCT) found from DFT calculation are tabulated in Table 2 along with experimental values. A comparison study of lattice parameters for both pure and doped BT between their theoretical and experimental values was found similar to other works. The resultant values of pure BT are so close to the experimentally obtained value. The calculated lattice parameter of BT has been optimized a = b = c = 3.979 Å which is almost equal to the theoretical value reported for pure BT^41^, and experimentally found 4.015 Å^42^. The difference in lattice parameter is about 0.04 Å that less than 1%. According to the calculated value, a satisfactory result has been found that is close to previous experimental data. Good accuracy in the calculation of lattice parameters can be an important factor for further calculation of structural calculation of any perovskite oxide. The calculated lattice parameter for doped BT ceramic was found in a range of 3.895–3.979 Å.

**Table 2:** Calculated lattice parameter and symmetry of pure BaTiO_**3**_ and Ca doped BaTiO_**3**_.

Composition	Symmetry Space	a_o_ (Å) This	a_o_ (Å) Cal.	a_o_ (Å) Expt.	V_o_ (Å^3^) This
BaTiO_3_	Pm3̅m	3.979	3.975^41^	4.015^42^	62.997
Ba_0.875_Ca_0.125_TiO_3_	Pm3̅m	3.961	–	3.0.962^43^	62.146
Ba_0.750_Ca_0.250_TiO_3_	P4/mm	3.944^a^	–	3.946^29^	61.334
		3.943^c^			
Ba_0.625_Ca_0.375_TiO_3_	Pm3̅m	3.929	–	–	60.652
Ba_0.500_Ca_0.500_TiO_3_	Pm3̅m	3.910	3.911^41^	–	59.777
Ba_0.375_Ca_0.625_TiO_3_	Pm3̅m	3.895	3.895^41^	–	59.091

*Cal.* = calculated value, *Expt.* = experimental value. Here, a and c are the lattice constant for the tetragonal (p4/mm) phase.

The lattice parameter of the cubic phase BT is decreasing with the Ca increasing due to the difference of ionic radii of Ba and Ca atom, where Ca^2+^ (radii = 1.34 Å), Ba^2+^ (radii = 1.64 Å) occupy the A site of the cubic BT ceramic. Due to the poor number of previous research on DFT calculation for multi-quantity Ca doped BT, a full comparison study between the theoretical and experimental calculation of BCT can’t be shown in Table 2. We notice symmetry space p4/mm found for Ba_0.750_Ca_0.250_TiO_3_ (x = 0.25). That means the crystal transformed into a tetragonal phase due to doping level x = 0.25 with lattice constant a = 3.944 Å and c = 3.943 Å. The reason behind phase change may occur due to the incorporation of a high concentration Ca atom that is dissimilar to the host atom. We noticed the lattice parameter and unit cell volume have lessened with a small fractional value after Ca doping. We found the tetragonal symmetry (P4/mm space group) for the crystal when we run the symmetry finding operation. In this investigation, all the characteristics have been carried out at room temperature. Yes, according to the literature it is true that phase transition occurred from tetragonal to cubic at room temperature, but phase transition also depends on other factors, such as doping concentration. If the doping concentration of Ca in BT ceramic get more than x = 0.2–0.25 then the phase occurs from the tetragonal phase to the cubic phase. So it is possible that for x = 0.25 the structure become unstable but for more than x = 0.25 structure of Ca doped BT becomes stable in the cubic phase.

### Electronic properties

**Figure 3:**
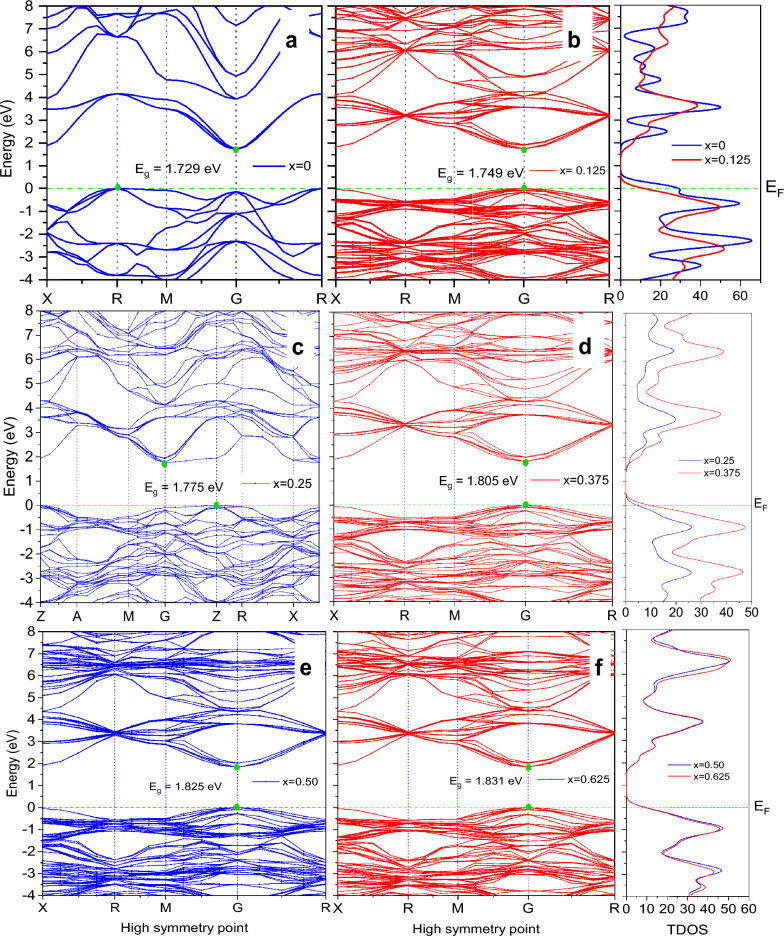
Band Structures with corresponding TDOS of Ba_1−x_Ca_x_TiO_3_ where (**a**) x = 0, (**b**) x = 0.125, (**c**) x = 0.25, (**d**) x = 0.375, (**e**) x = 0.50 and (**f**) x = 0.625.

The study of electronic band structure provides useful information to realize paraelectricity and ferroelectricity including semiconductor behavior. Electronic band structures have been calculated by using the GGA-WC approaches and figured out in this paper as a part of electronic properties for both pure and doped BT along with high symmetry directions. This band structure provides a realization of the electronic structure of optimized cubic BT perovskite and also how it changes due to doping. Figure 3 is illustrated the band structure of pure BT and every Ca doped BT. The first figure indicating Fig. 3a is for pure BT and the rest of all is sorted following doping percentage. The maximum level of the balance band at R-point and the minimum level of the conduction band at the G-point provide evidence of an indirect bandgap (BG) material of magnitude 1.729 eV. This value was found also in previous research that proves the good accuracy of our theoretical work.

**Figure 4:**
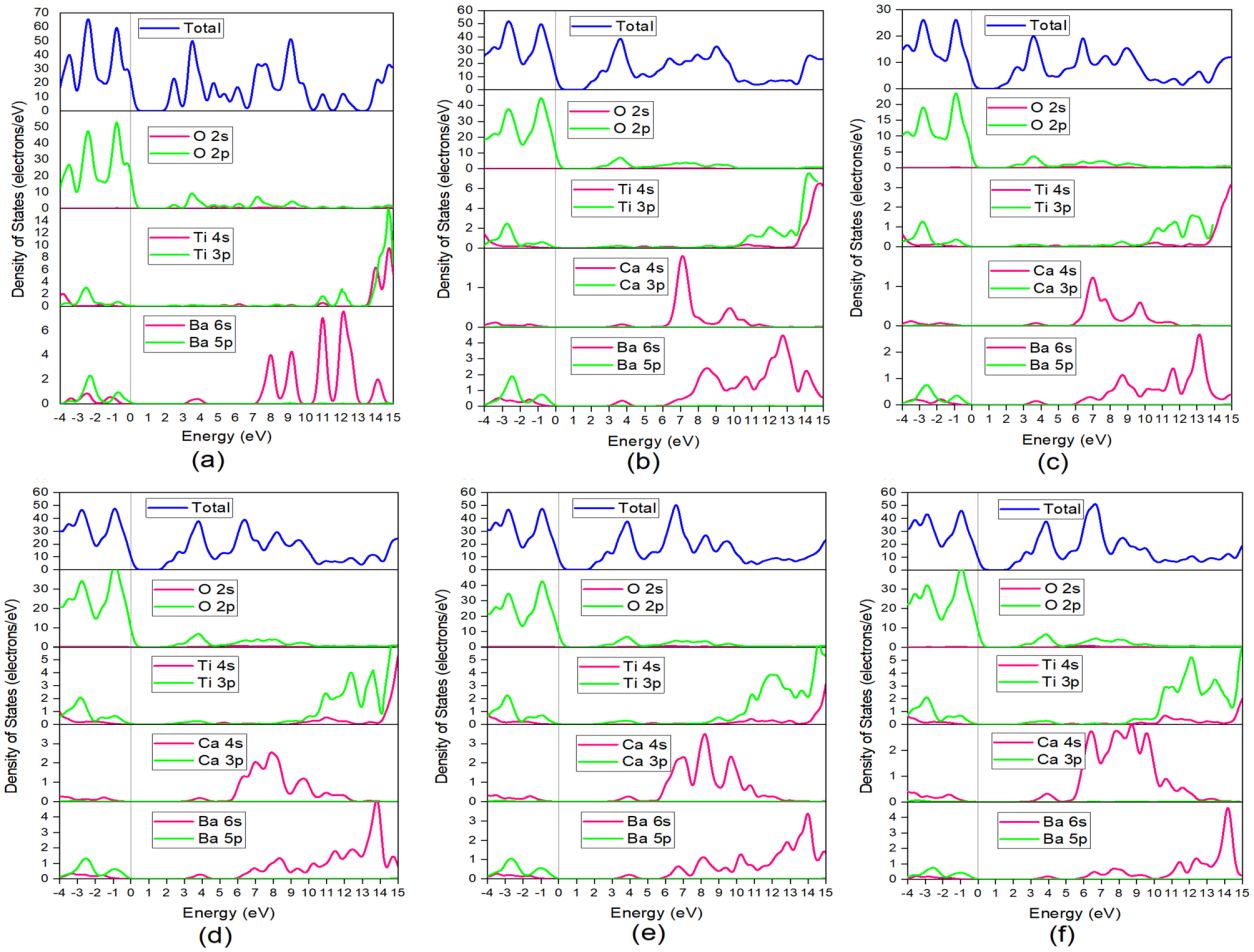
Partial density of states (PDOS) and Total density of states (TDOS) of Ba_1−x_Ca_x_TiO_3_ where (**a**) x = 0, (**b**) x = 0.125, (**c**) x = 0.25, (**d**) x = 0.375, (**e**) x = 0.50, (**f**) x = 0.625.

All the calculated electronic bandgap (E_g_) and comparative studies with theoretical and experimental values are listed in Table 3. It is clear from Fig. 3 that the expansion of valance bands does not exceed the 0 eV though valance shells are extended to several eV in the negative portion. The green line at the 0 eV energy level represents the Fermi energy level (E_F_). We found a slight raise of bandgap after doping Ca (x = 0.125) have noticed in Fig. 3 with direct bandgap at G-point and the value is about 1.749 eV. Though in most cases Ca doped BT shows direct bandgap, the exceptional case has shown when x = 0.25. By doping the Ca atom, we found a direct bandgap, as indicated in the figure. A direct bandgap is needed for a crystalline material to be a credible tool for photovoltaic, optoelectronic, and photothermic applications in general^43^. Due to phase transition, we found a P4/mm space group including an indirect bandgap with a value of 1.775 eV when doping was performed with 25% Ca (x = 0.25) into BaTiO_3_. That is an interesting part that arises more research questions. This phenomenon may cause for some reasons. Such as, at low doping concentrations in a semiconductor, atomic (i.e. localized) states are introduced inside the gap^46^. These atomic states overlap and generate additional impurity bands as the doping concentration is increased. That may cause the formation. We notice a slight increase of bandgap value due to every doping percentage respectively as a direct bandgap found for the incorporation of Ca atom into BaTiO_3_, further experimental and theoretical research scope will have arisen. Generally, shorter bonds result in electrons being more closely linked to the atom and requiring more energy to be removed, increasing the bandgap. As a result, bigger bandgaps are correlated with smaller lattice constants. The Ba atom has more metallic properties than Ca atom, so doping of Ca replacing the Ba atom increases the bandgap^47^. The Obtained electronic bandgaps E_g_ of this study are listed in Table 3 along with other theoretical and experimental data. The lack of information on theoretical and experimental values in Table 3 is due to little research work published on this topic. After all, our research aim was to observe the variance of bandgap in BaTiO_3_ due to Ca-doping and to avoid the bandgap error for the GGA method. Figure 4 shows the partial density of states (PDOS) and total density of state (TDOS) for pure BT and Ca doped BT given sequentially with respect to doping percentage.

**Table 3:** Comparison tabulation of bandgap reported in the literature and Calculated bandgap E_g_ of pure and Ca doped BaTiO_3_.

Ba_1−x_Ca_x_TiO_3_ x	Parameter		
	Obtained	Literature	Experimental
	Bandgap E_**g**_ (eV)		
0	1.729	1.56^44^	3.1^45^
0.125	1.749	1.91^29^	–
0.250	1.775	1.86^29^	–
0.375	1.805	–	–
0.500	1.825	–	–
0.625	1.831	–	–

The partial density of states showed that atomic orbitals were involved in the total density of states plot. The valance band (VB) of BT is formed of Ba 5s, Ba 5p, Ti 3s, Ti 3p, Ti 3d, O 2s, and O 2p state. In contrast, the conduction band (CB) is composed of Ba 6s and Ti 4s state in pure BT. From Fig. 4 of PDOS, VB of pure system of x = 0.0 is mainly formed due to O 2p orbital with a tiny contribution of Ba 5p and Ti 3p states. Here O-2s states dominate the core electrons, which are situated about 1–3 eV region. On the other side, Ba 6s and Ti 3p state are leading the CB in pure BT and little contribution of Ca 4s state after doping is noted as it has shown. With a vertical dotted line in the figure, the Fermi energy level is shown by a zero point at the energy level. Close to the Fermi level, the maximum VB and minimum CB are relatively acute. This is comparable to the experimental result^48^ of optical measurements of BaTiO_3_ with a very strong absorption edge. When Ca incorporated with respect to Ba following the formula Ba_1−x_ Ca_x_TiO_3_, it contributed to the Conduction band (CB) with Ba 6s state but the bandgap did not change dramatically since Ba and Ca are both alkaline earth metal and have identical chemical properties. After incorporating Ca in BT, Ba 6s lies at 12–13 eV as before but density peaks have decreased a bit. Further increasing the doping content leads to a little shift of the Ba 6s state to the right and lessens the density peak. Respectively Ti 3s state lies at 16 eV with respect to Ba but after doping it has moved to the left slightly. The orbitals in the valence band are organized in order of highest to lowest involvement for both cases. There are some co-factor control decrements or increments of the bandgap energy. The existence of the dopant atom’s orbitals in the VB or CB may cause the energy levels to change due to hybridization with the current orbitals. After increasing the doping level, the electron density of Ca was growing inside the crystal. Any shifting of Fermi energy level to the conduction band did not found nor was free-electron splitting due to doping. On the contrary band, the gap increased as Ca does have not metal-like properties. In the band structure diagram if the VB crosses the Fermi level it is difficult to calculate the band gap value. This Fermi level is within the conduction band in the case of a degenerate level of doping, this effect is known as Moss–Burstein effect^49^. The Moss–Burstein effect does not occur in our study. The Moss–Burstein effect occurs when the absorption edge of a semiconductor is pushed to higher energies as a result of certain states close to the conduction band being occupied and when the electron carrier concentration exceeds the CB edge of DOS. This variation may cause for using different functions in the simulation package and also for using different doping content of a degenerate level of doping.

### Optical properties

In most cases, the optical behavior changes with the modification internal microstructure of materials. This modification can be performed either by applying physical strains or a chemical doping process^50,51^. Therefore, materials may show improved optical properties with appropriate dopant or physical changes. Investigation of the optical properties of a perovskite material is a fundamental need to realize its suitability for uses in photorefractive, optoelectronics and solar cell (Photovoltaic) applications and others^52^. The influences of Ca dopant on the optical properties of BT ceramic were calculated using GGA-WC functional approach via CASTEP. In this first principle investigation, frequency-dependent optical properties such as Absorption coefficient, Reflectivity, Conductivity, Refractive index, Extinction coefficient, Dielectric function and Loss function were investigated at the energy range of 0–30 eV and described with separate sections. As we find from the wavelength spectrum almost 0–2.75 eV energy is referred to as the infrared region, whereas 2.75–5.2 eV referred to the visible region and more than 5.2 eV but less than 30 eV lies under the UV region of light. At first, we calculated the optical properties of pure BaTiO_3_ ceramic and then compared them with Ba_1−x_Ca_x_TiO_3_, where x = 0.0, 0.125, 0.25, 0.375, 0.50 and 0.625.

#### Absorption coefficient

**Figure 5:**
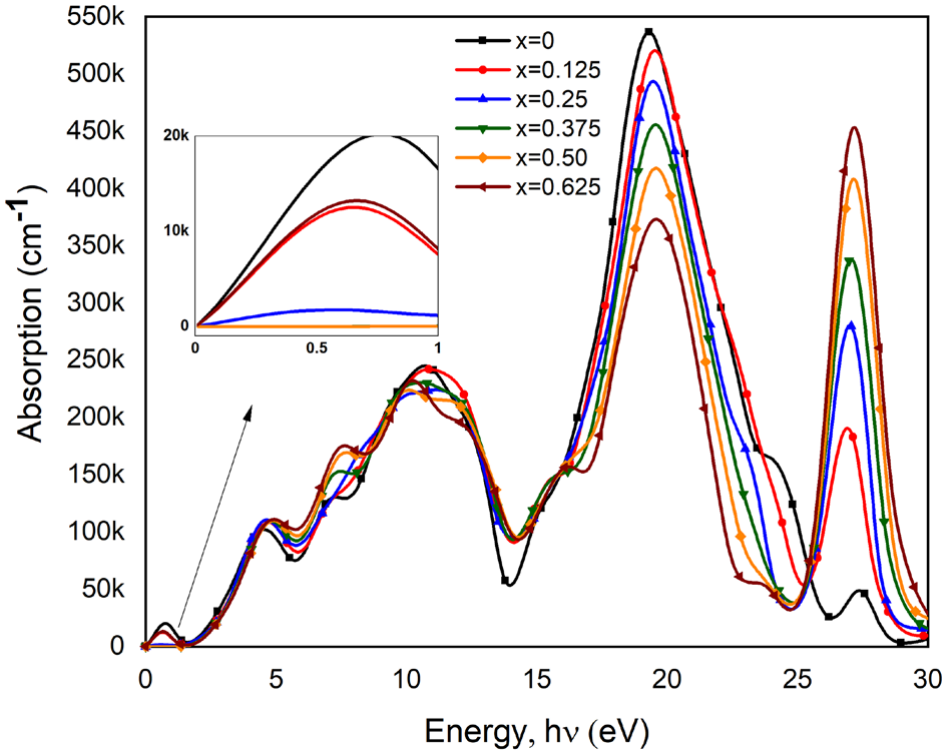
Frequency dependant Absorption coefficient as a function of photon energy of pure and Ca doped BaTiO_3_.

The absorption Coefficient provides an assumption of optimum Solar Energy Conservation efficiency for solar cell applications. In determining the performance of solar cells, the optical absorption coefficient is an essential parameter^53^. The calculated absorption spectra illustrated in Fig. 5, show a lower value at the visible light region rather than a prominent peak found for pure BT in 20 eV at the UV (ultraviolet light) region. UV absorption is an important tool and it has many uses in some applications where BaTiO_3_ can be used as found a good absorption coefficient in the UV light energy region. But less efficient than solar cells both pure BT and Ca doped BT. The absorption coefficient defines how far light of a specific wavelength may reach into a substance before being absorbed. As light penetrates through the materials, electrons absorbed energy and travel from the valance band to the conduction band. This absorbed light energy introduces the absorption peak. Absorption reduces in a small amount after doping Ca due to the reduction of BG (Bandgap) and transferring Indirect-BG to Direct-BG probably. Highly localized interband transition is responsible for the increment of absorption spectra. A lower absorption peak is found at the lower energy level whereas a higher peak is at the higher energy level. At the lower energy region, the absorption is noticed higher for pure BT ceramic but at the higher energy region. For the value of x = 0.625, the material shows the highest absorption. Multiple absorption peaks may belong to the electronic transition between Ti-d to Ca-d states^30^.

#### Reflectivity

**Figure 6:**
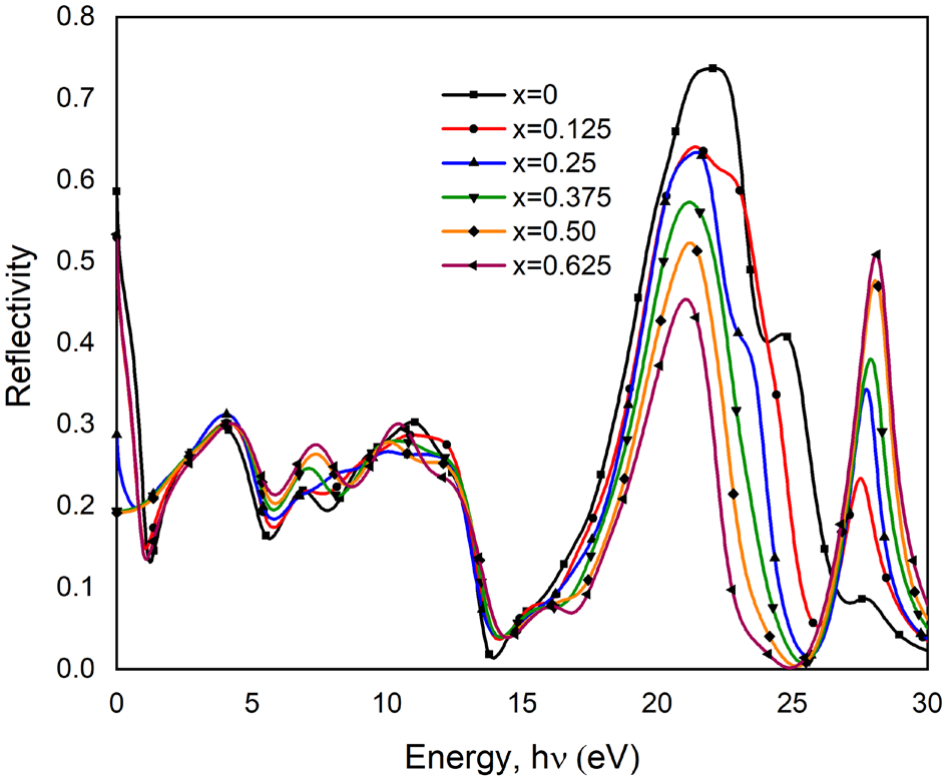
Reflectivity, $R\left(\omega \right)$ versus photon energy of pure and Ca doped BaTiO_3_.

Corresponding to the absorption spectroscopy, the reflectivity $R\left(\omega \right)$ spectra in Fig. 6 show the reflection of light is lower where the absorption is higher. Reflection occurs on the material’s surface and, in the case of light-diffusing materials, also in the volume. At the 0 eV region, reflection is maximum for pure BT and minimum for x = 0.50 material due to wider electron density after increasing doping content. Higher reflectivity is found for high wavelengths. This feature may be utilized in UV ray applications. The curves show that reflectivity peaks occur at 21.8 eV and 27.2 eV for pure BT. These peaks are due to the interaction between O-2p and Ti-3d states^28^. Absorption in the visible light area is not satisfying rather to the UV region. Absorption peaks are not in the expected region following the figure rather reflectivity property is seen. That’s why that material cannot be recommended strongly for solar cell applications. To ensure that the material has good optical characteristics, optical-bandgap needs to include under investigation that found from optical properties analysis through GGA-WC function. The electronic band gap is the barrier for producing an electron–hole pair that is not bonded together, while the optical bandgap is the threshold for photon absorption^54^. Here, Table 4 include calculated optical bandgap data that shows the optical bandgap for both doped and pure BT compound. Optical bandgap found 2.06 eV for pure BT, 1.98 eV for x = 0.125, 1.77 eV for x = 0.25, 2.01 eV for x = 0.375, 2.04 eV for x = 0.50 and 2.05 eV for x = 0.625. The excited electrons absorb energy injected into the conduction band from the valance band. A lower energy gap requires for a good photocatalysis material. Here, the minimum optical bandgap is noticed for x = 0.25 which can be suggested as a good photorefractive material.

**Table 4:** Optical bandgap of undoped and Ca doped BaTiO_3_.

Sample: Ba_1−x_Ca_x_TiO_3_						
Doping content	0	0.125	0.25	0.375	0.50	0.625
Bandgap (eV)	2.06	1.98	1.77	2.01	2.04	2.05

#### Complex refractive index

**Figure 7:**
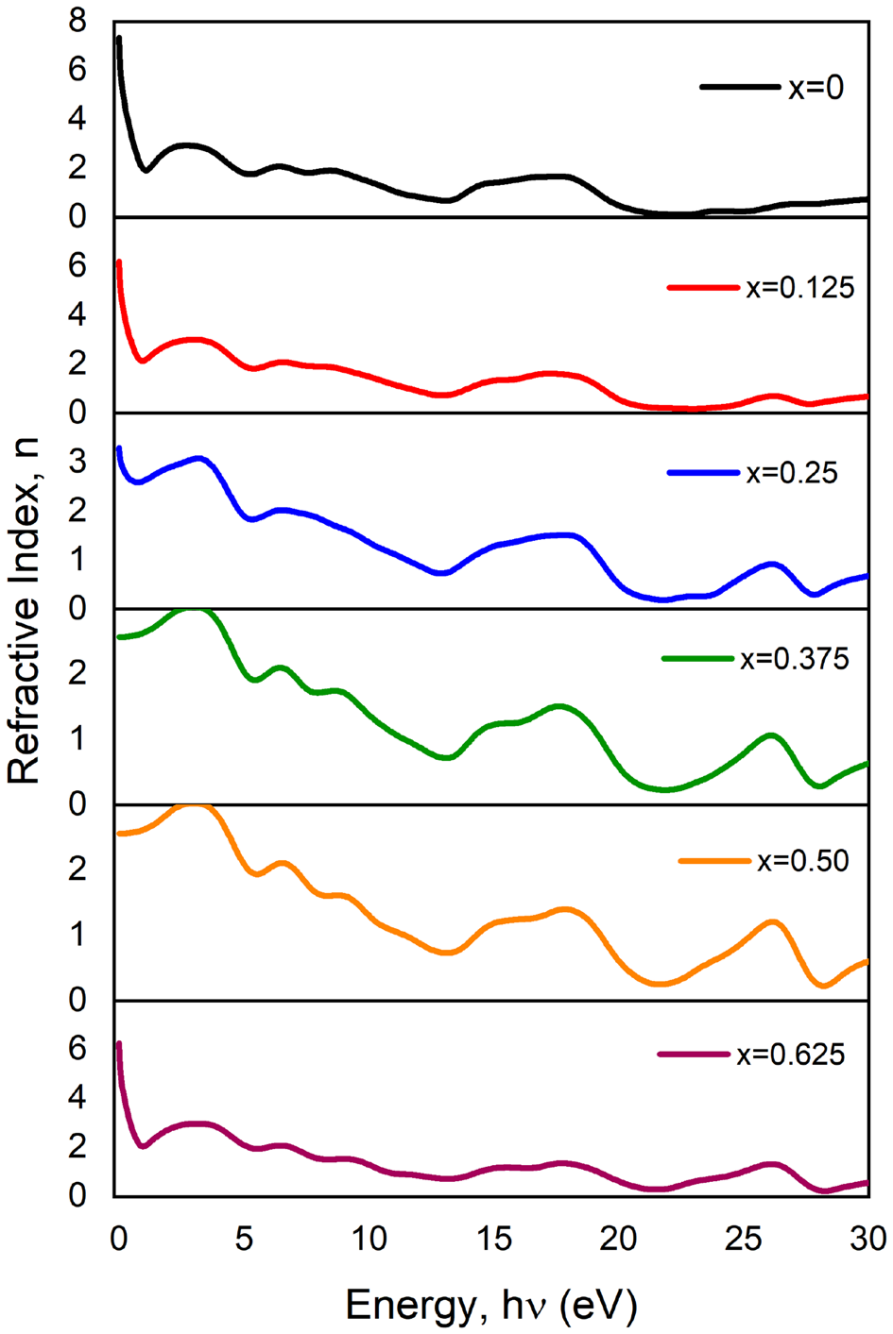
Variation of Refractive Index, $n\left(\omega \right)$ versus photon energy of pure and Ca doped BaTiO_3_.

**Figure 8:**
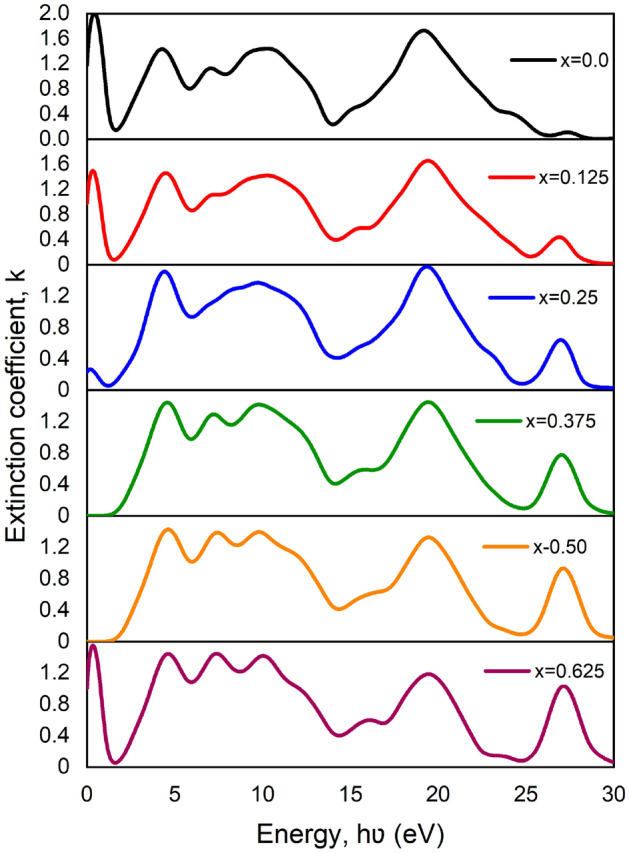
Variation of extinction co-efficient, $k\left(\omega \right)$ versus photon energy of pure and Ca doped BaTiO_3_.

A graphical view of the frequency-dependent refractive index, $n\left(\omega \right)$ and extinction co-efficient, $k\left(\omega \right)$ is given in Figs. 7 and 8 respectively. Both of them come from the complex equation^55^ N = n + ik, where ‘n’ is denoted for the refractive index and ‘k’ is for the extinction coefficient. This complex equation consists of real and imaginary parts. Here extinction co-efficient plays the role of an imaginary part that depends on energy. The term “extinction coefficient” applies to several distinct measurements of light absorption in a medium. The extinction coefficient is a measurement of how well a material absorbs light at a specific wavelength. Contrariwise, when light enters a substance, the refractive index affects how much the path of light is twisted, or refracted. In the plotted figure, the maximum refractive index found for pure BT is 7.2 and this value is decreasing according to doping the percentage. But another prominent value was found suddenly for x = 0.625. This is due to a drop in absorption coefficient after doping and an increase in transmission with photon energy^56^. On the other side, the highest value of the extinction coefficient of pure BT is 2.0 at 0 eV energy. It’s also decreasing like the refractive index according to doping percentage except for x = 0.625. Generally, the refractive index (n) has a decreasing trend with an increase in photon energy. Here ‘k’ indicates the terms annihilation of energy within the system. It is also related to absorption energy^57^.

#### Optical conductivity

**Figure 9:**
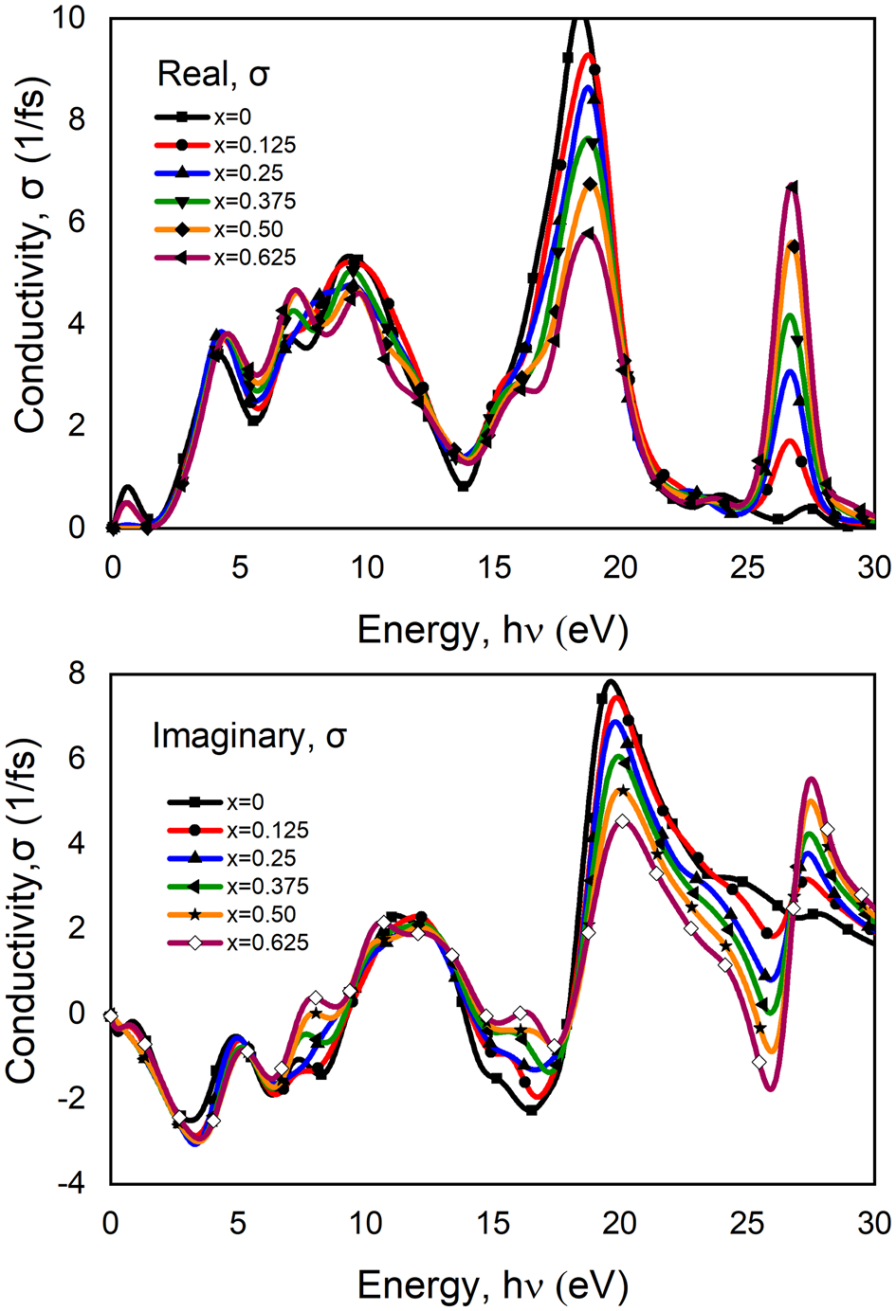
The real and imaginary part of conductivity concerning photon energy of pure and Ca doped BaTiO_3_.

Optical conductivity is another part of optical properties that provides an idea of conduction when light falls on it, a powerful tool also to measure the electronic state of a material. The expansion of electrical transmission to high (optical) frequencies is known as optical conductivity^58^. In the presence of an alternating electric field, the phrase “optical conductivity” refers to electrical conductivity. While the optical conductivity of insulators is always limited at specific frequency intervals above the optical gap, the static electrical conductivity is vanishingly tiny. An investigation of the frequency-dependent optical conductivity of pure and Ca doped BT has shown in Fig. 9. The dielectric function, which is an extension of the dielectric constant to variable frequencies, is strongly linked to optical conductivity. The following equation^56^ connects the complex optical conductivity (σ*) to the complex dielectric constant (ε*).

1 $$\upsigma_{{1}} = \, \upomega \upvarepsilon_{{2}} \upvarepsilon_{0} {\text{ and }} \upsigma_{{2}} = \, \upomega \upvarepsilon_{{1}} \upvarepsilon_{0} ,$$

where ω is denoted for angular frequency, ε_1_ and ε_2_ for real and imaginary dielectric function and ε_0_ for free-space dielectric constant respectively. Here presented both real and imaginary parts found from DFT calculation. Higher losses and higher absorption are associated with a higher real portion of conductivity when given a wave of the same frequency. Considering the real part, the optical conductivity for both pure barium titanate and Ca doped barium titanate shows a good value compared to low energy rather than some other perovskite material. A noticeable point is that Ca doped BT shows high absorption rather than pure system though pure system shows a prominent value at high energy region. Conductivity fluctuates its value in a small amount after doping Ca ions which can be explained by the bandgap energy of materials. For those features, it can say that both pure and doped BT is a good candidate for photorefractive and optoelectronic devices. But the maximum conductivity is 10, which has shown high energy in the UV region by pure BT compared to doped impure BT crystal. The second conductivity peak is in the 25–30 eV region. The conductivity graph is relating to absorption spectra, which look close to the absorption graph. Where absorption is high, conductivity is also high.

#### Dielectric function

The most important behavior of optical properties can be investigated by the dielectric function along with other parameters such as reflectivity, refractive index and extinction Coefficient, etc. Those are found by following the equation^59^ given in DFT for both pure and doped barium titanate. Therefore, the real and imaginary part of the dielectric function is the most important parameter from which we can find the other optical properties. Here is the mathematical expression for reflectivity $R\left(\omega \right)$ in Eq. (1), Loss function $L\left(\omega \right)$ in Eq. (2), extinction coefficient $k\left(\omega \right)$ in Eq. (3), refractive index $n\left(\omega \right)$ in Eq. (4), complex refractive index $\mathrm{N}\left(\upomega \right)$ in Eq. (7) in terms of the dielectric function^59^.

2 $$R\left(\omega \right)=\left[\frac{\sqrt{\upvarepsilon \left(\upomega \right)} -1}{\sqrt{\upvarepsilon \left(\upomega \right)}+1}\right],$$

3 $$L\left(\omega \right)=\frac{{\varepsilon }_{2}\left(\omega \right)}{{\varepsilon }_{1}{\left(\upomega \right)}^{2}+{\varepsilon }_{2}{\left(\upomega \right)}^{2}} ,$$

4 $$k\left(\omega \right)=\frac{1}{2}[\sqrt{{\varepsilon }_{1}{\left(\upomega \right)}^{2}+{\varepsilon }_{2}{\left(\upomega \right)}^{2}}-{\varepsilon }_{1}\left(\upomega \right){]}^\frac{1}{2},$$

5 $$n\left(\omega \right)=\frac{1}{\sqrt{2}}[\sqrt{{\varepsilon }_{1}{\left(\upomega \right)}^{2}+{\varepsilon }_{2}{\left(\upomega \right)}^{2}}-{\varepsilon }_{1}\left(\upomega \right){]}^\frac{1}{2},$$

6 $$r\left(\omega \right)=\frac{n+ik-1}{n+ik+1},$$

7 $$\sqrt{\upvarepsilon \left(\upomega \right)}=n\left(\upomega \right)+\mathrm{ik}\left(\upomega \right)=\mathrm{N}(\upomega ) ,$$

8 $${\varepsilon }_{1}\left(\upomega \right)={n}^{2}-{k}^{2},$$

9 $${\varepsilon }_{2}\left(\omega \right)=2nk.$$

**Figure 10:**
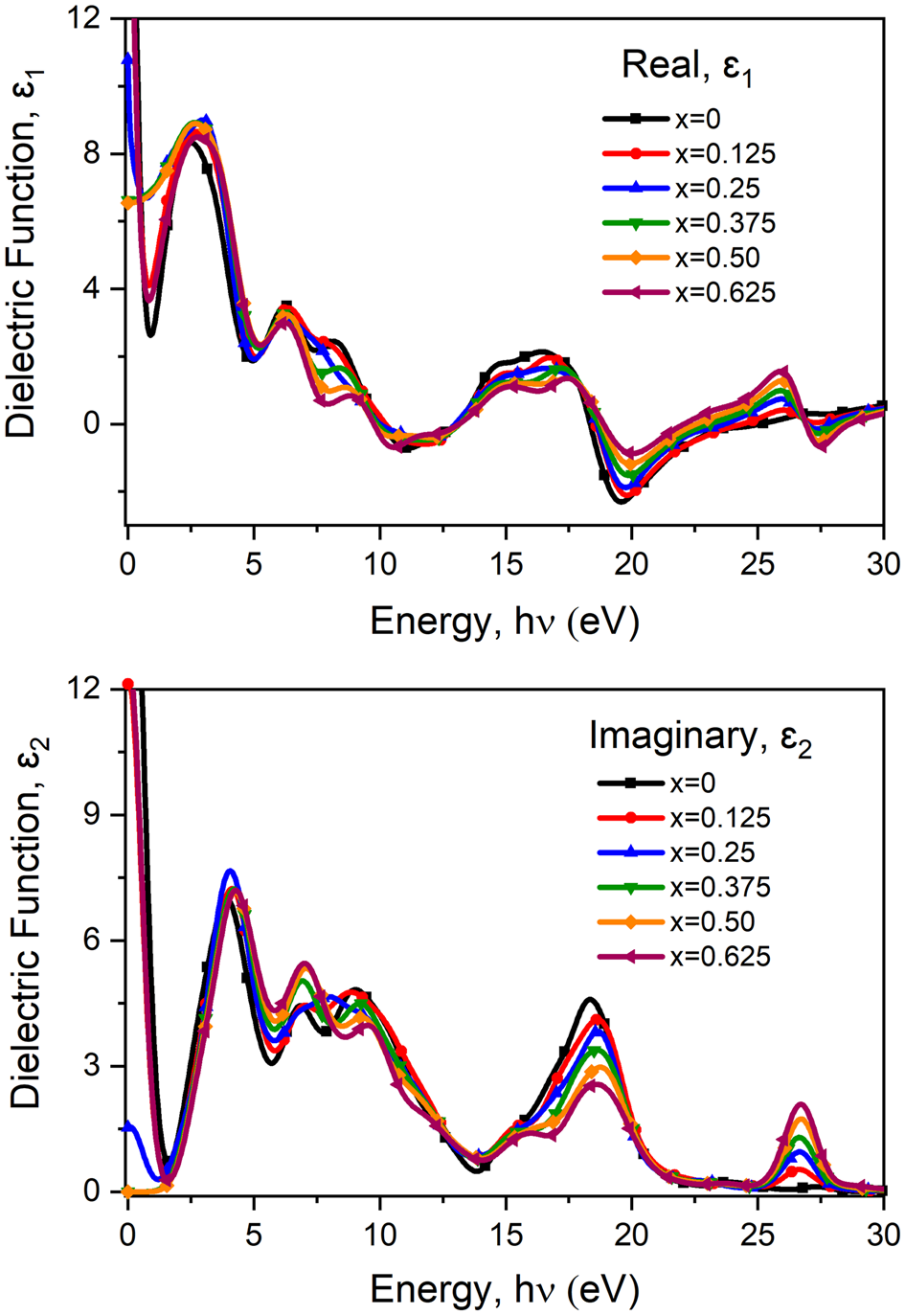
The real and imaginary part of dielectric function with respect to a photon energy of pure and Ca doped BaTiO_3_.

The calculation for frequency-dependent dielectric function which is so significant for optical properties has been done with both real and imaginary parts of the dielectric function following equation Eq. (9)^60^

10 $$\upvarepsilon \left(\upomega \right)={\upvarepsilon }_{1}\left(\upomega \right)+i{\upvarepsilon }_{2}\left(\upomega \right) ,$$

where ${\upvarepsilon }_{1}\left(\upomega \right)$ is the real part of the dielectric function and ${\upvarepsilon }_{2}\left(\upomega \right)$ is the imaginary part of the dielectric function. The equation of dielectric function $\upvarepsilon \left(\upomega \right)$ expressed in Eq. (9) depends on both electron interaction and photon energy which is responsible for the inter-band and intra-band transition. Rizwan et al*.*^30^ says “${\upvarepsilon }_{2}\left(\upomega \right)$ can be obtained from the electronic band structure directly and ${\upvarepsilon }_{1}\left(\upomega \right)$ by using Kramer–Kroning dispersion relation. As the energy of incident radiations increases, ${\upvarepsilon }_{1}\left(\upomega \right)$ which corresponds to the polarization of material approaches its maximum value”^30^. Figure 10 showed for the real part ${\upvarepsilon }_{1}\left(\upomega \right)$ which represents polarization and the imaginary part ${\upvarepsilon }_{2}\left(\upomega \right)$ represented for loss of energy in the medium. In other words, the real part of the dielectric function indicates the ability of the material to store charge and the imaginary part of the dielectric function indicates the ohmic resistance of the material that’s expressed as loss in the material. The figure of ${\upvarepsilon }_{1}\left(\upomega \right)$ shows a higher value at 0 eV except for x = 0.50 which means a potential dielectric material for both BT and BCT is for their satisfying value of dielectric constant. For x = 0.50 material shows zero value at 0 eV. Whereas, the figure of ${\upvarepsilon }_{2}\left(\upomega \right)$ through other curves shows a peak. Each of the figures shows the three prominent peaks. For the real part of pure BT three prominent peaks at 2.80 eV, 16.31 eV and 25.88 eV. But for the doped BT ceramic no significant change was not found except prominent peaks shifted a little towards the right of the energy region. The same results occur for the imaginary part of the dielectric function. But they are considered a good candidate for some optoelectronic devices where dielectric material is needed.

#### Loss function

**Figure 11:**
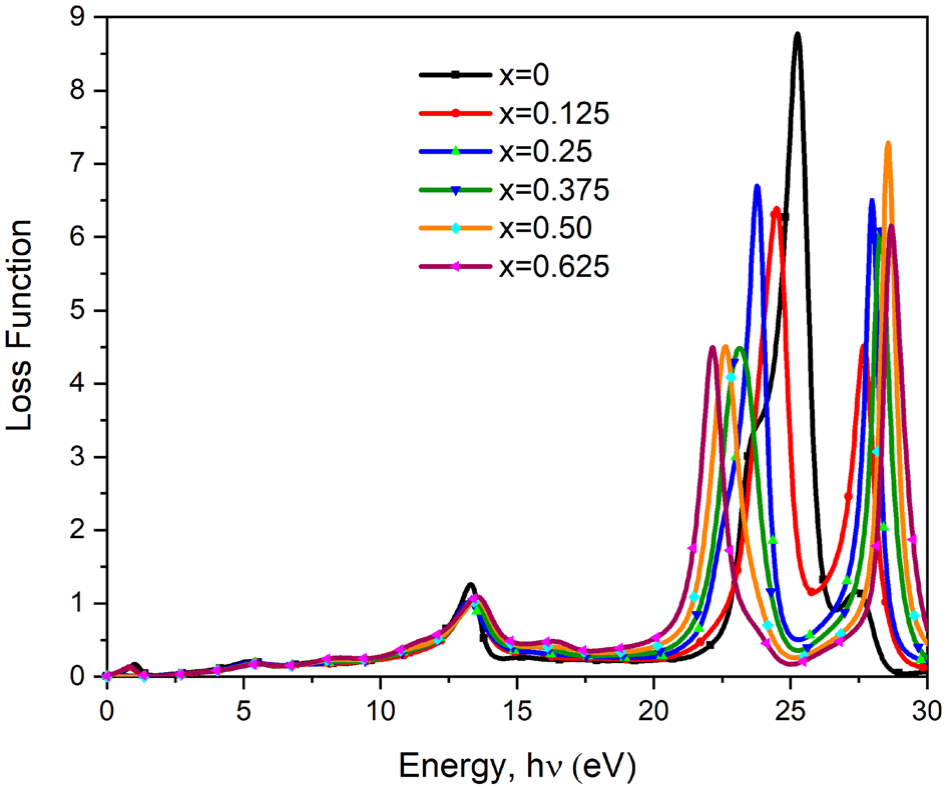
The loss function versus photon energy of pure and Ca doped BaTiO_3_.

The loss function $L\left(\omega \right)$ for both pure and doped BT have shown in Fig. 11. By the figure, the energy area where electrons aren’t bounded in their lattice site but exhibit plasma frequency, whenever expounded in light, is called the loss function of energy an atom^45^. The highest peak for loss function was found for the pure system. That peak shifted towards high energy relating to ${\upvarepsilon }_{2}\left(\upomega \right)$ as shown in the figure. The loss function peaks above 20 eV represent the transition from the bottom of the valence band to CB, namely from O-2p to Ti-3d states^30^.

The responsible equation of loss function $L\left(\omega \right)$^28^ have presented below in Eq. (11).

11 $$L\left(\omega \right)=\left[\frac{{\upvarepsilon }_{2}\left(\upomega \right)}{{\varepsilon }_{1}{\left(\upomega \right)}^{2}+{\varepsilon }_{2}{\left(\upomega \right)}^{2}}\right].$$

Here, all obtained figures of optical properties after DFT calculation ranges between 0 and 30 eV as shown. As pure BT and Ca doped BT are equivalent perovskite material, major changes in properties were not found following the calculation though slight changes were noticed in some cases which leads to the doped material to use as good photorefractive material.

### Mechanical properties

In this section, we determine and explain the mechanical stability and different mechanical properties of Ba_1−x_Ca_x_TiO_3_ where x = 0.125, 0.25, 0.375, 0.500, 0.625. Measured data of elastic constants and Cauchy pressure are included in Table 5. It is crucial to understand how elastic constants respond to various doping ratios^60^. Important background information on phonon spectra, machinability interatomic bonding, equations of state, and interatomic potentials may be gleaned from a material’s elastic characteristics. Combining the lattice symmetry with Hook’s rule yields just three distinct constants (C_11_, C_12_, and C_44_) for cubic compounds^61^. For a compound to be mechanically stable, its elastic constants must comply with the Born stability criteria C_11_ > 0, C_44_ > 0, C_11_ − C_12_ > 0, and C_11_ + 2C_12_ > 0^62^. According to the data of elastic constants, both doped and undoped compounds can be said to be mechanically stable since they fulfil the conditions of the born stability criteria.

**Table 5:** Various elastic constant of Ca doped BaTiO_3_ under first principle calculation.

Content	C_11_	C_12_	C_44_	C_12_–C_44_
0	202.89	75.59	107.60	− 32
0.125	322.06	111.2	116.96	− 5.76
0.25	284.67	96.19	110.1	− 13.91
0.375	295.41	99.58	111.69	− 12.11
0.500	344.58	109.55	110.63	− 1.08
0.625	334.395	109.18	111.23	− 2.05

**Figure 12:**
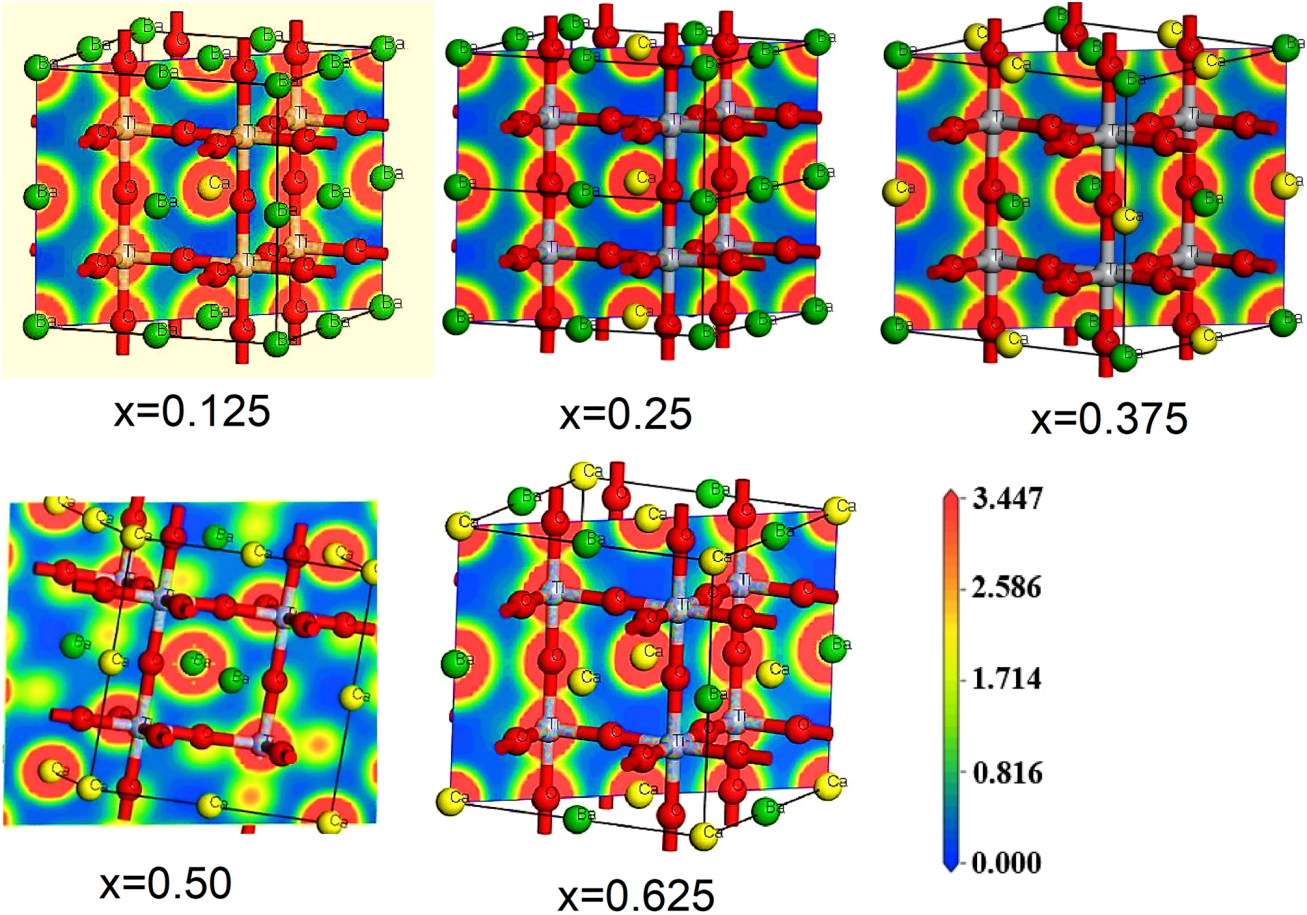
Charge-density distributions of all Ca doped BaTiO_3_ in (110) surfaces.

An increase in C_11_ elastic constant following doping is indicative of a stiffening of the material along the crystallographic direction under study. Along that direction, its resistance to being squashed or stretched increases. For this phenomenon, the structural distortion of the lattice lessens and stability is boosted. At this value of x = 0.25 there is a sudden drop in the elastic constants as the crystallographic phase changes at this value. Cauchy pressure (C_12_–C_44_) has been proposed in the literature as a means of describing bonding type. If the Cauchy pressure is positive, then the ionic bond is being expressed; otherwise, the covalent bond is being expressed by the negative value^61^. More directed bonding occurs at lower Cauchy pressures, whereas metallic bonding predominates at higher pressures. Also, those modified compounds exhibit brittle behavior following elastic constant data. Since there is no metal phase in our investigated material, the presence of covalent bonds is observed here. We analyzed electronic charge density mappings in order to gain a better understanding of the bonds that exist in our compounds and the distribution of charge around the atoms. Figure 12 shows the results of the study into the charge density distribution of all Ca doped BaTiO_3_ along (110) crystallographic plane that will help to visualize the co-valent bonds. The charge density is shown by the colored scale on the right side of the graph, where red signifies high density and blue shows low density accordingly. It suggests that strong covalent connections are formed between Ti–O.

### Thermodynamic properties

**Figure 13:**
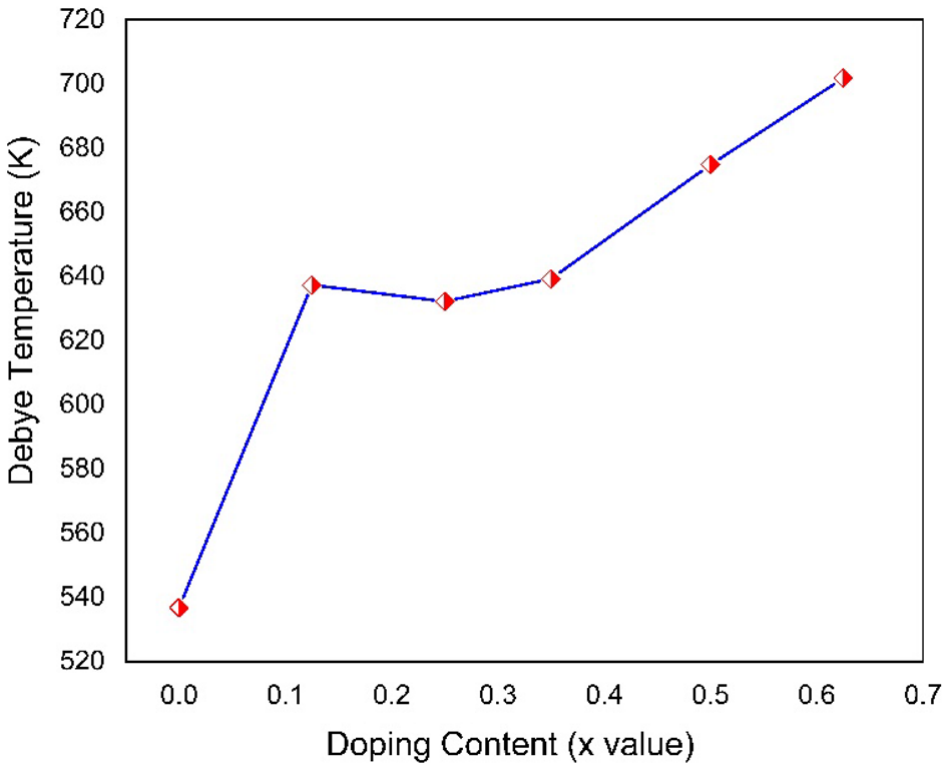
Variation of Debye Temperature as a function of doping content.

Studying the development of solid-state physics as well as thermodynamic chemistry requires an understanding of the importance of thermodynamic properties. The temperature at which all atomic modes of vibration are active is known as the Debye temperature^63^. At the Debye temperature, solid lattice vibrations (phonons) become evident. It helps determine how vibrational modes affect a material's physical properties. The variation of Debye Temperature with doping content is shown graphically in Fig. 13. A higher Debye temperature indicates that the covalent bonds are becoming more potent and the compound is becoming less pliable when the doping content is increased^64^. Calcium doping increases the atomic mass of barium titanate which increases the debye temperature as it is proportional to the square root of atomic mass. Moreover, it can be seen in the mechanical properties section that Ca doping increases mechanical stability and reduces the structural distortion of the lattice^65^. Stable crystals have high vibrational energy due to which the debye temperature increases.

## Conclusion

In this study, we have characterized the structural, electronic and optical properties of pure and Ca doped BaTiO_3_ theoretically using first-principle DFT calculation with the GGA-WC function mentioned above. The obtained results of structural properties indicate the successful characterization of doped BT solutions. The results are also in good agreement with the theoretical values in the literature. Structural properties like lattice parameters, volume and symmetry space were successfully characterized. Moreover, Bond lengths, bond angles of atoms and off-center of Ti atoms were measured after geometrical optimizations. The band structure along with TDOS and PDOS has been calculated for 2 × 2 × 2 supercell of BCT. Importantly, the doped BT solution has the direct bandgap at G-point except for x = 0.50 and the bandgap energy E_g_ has increased with the increasing Ca ion. Optical behavior has been investigated at the energy range of 0.30 eV. All the optical properties are well correlated with the electronic and structural parameters. Reflectivity, Refractive index (n) and Dielectric constant (ε_1_) were found lower for doped BT than pure BT where the absorption peak shifted higher to higher energies after doping. The prominent absorption peak at the UV light energy region and the obtained optical energy concludes that the doped BT solution is a suitable candidate for photorefractive and optoelectronic devices.

## Data Availability

The Data that support the findings of this study are available from the corresponding author upon reasonable request.
